# Substance use is associated with worse mental health and altered resting state functional connectivity in female university athletes at baseline: A pilot study

**DOI:** 10.1371/journal.pone.0253261

**Published:** 2021-06-17

**Authors:** Alyssia Wilson, Kristina Gicas, W. Dale Stevens, Lauren Sergio, Magdalena Wojtowicz

**Affiliations:** 1 Department of Psychology, York University, Toronto, Ontario, Canada; 2 School of Kinesiology, York University, Toronto, Ontario, Canada; Boys Town National Research Hospital, UNITED STATES

## Abstract

University athletes are at high risk for both substance use and mental health problems. This study examined associations between substance use, mental health symptoms, and the resting state functional connectivity (rsFC) of key neural regions involved in self-monitoring and emotional regulation in a sample of female varsity athletes. 31 female university athletes completed measures of substance use, mental health symptoms, and underwent functional MRI scans during the pre-season. Athletes who were substance users had higher symptoms of depression than non-users (p = 0.04; Hedge’s g = 0.81). RsFC differences were observed between users and non-users in orbital frontal cortex (OFC) and bilateral hippocampal seeds, and negative associations between depression symptoms and rsFC in the left hippocampus and posterior cingulate cortex were observed in cannabis users. In female athletes, substance use is associated with greater self-reported depression symptoms and altered rsFC in self-monitoring and emotional regulation regions of the brain.

## Introduction

Despite being targeted for substance-intervention and education, university athletes are more likely to use substances than their non-athlete peers [1]. In particular, athletes are more likely to engage in risky substance use behaviours, such as regular heavy alcohol use [2] and are at an increased risk for using other substances such as cannabis [3]. Team bonding, normative views of substance use, and increased pressures have been suggested to contribute to high use of substances among athletes [2].

In addition to being more susceptible to risky substance use behaviours, university athletes experience mental health problems at a similar rate or higher than the general population [4]. Athletes experience not only the same academic pressures experienced by all students but are also exposed to unique mental health risk factors including sports injury, performance pressure, overtraining, and conflict with coaches, when compared to their non-athlete peers [5]. Evaluating the associations between mental health and substance use is particularly important for young women, who are more likely to experience symptoms of anxiety and depression compared to males in general [6]. Specifically within the athlete population, females are 1.8 times more likely than males to endorse clinical-level symptoms in addition to reporting more severe symptoms [7].

There is evidence of overlapping brain-behaviour relationships between emotional regulation, substance use, and mental health. Differential activity in brain areas involved in self-monitoring and emotion regulation (e.g., the amygdala, prefrontal cortex, orbital frontal cortex; OFC, anterior and posterior cingulate cortices, and inferior parietal lobule) have been associated with both risky substance use [8,9] and mental health disorders [10,11]. Altered rsFC in depression is associated with inadequate reappraisal of emotions, increased rumination, and increased use of expressive suppression as a coping strategy [12]. Individuals with anxiety have been found to also have difficulty with adequate use of emotional regulation strategies [13].

Appropriate use of emotional regulation strategies is essential for decision making, and dysregulation of the neural processes underlying this function can affect social decision making [14]. Several key brain regions and neural networks have been implicated in supporting emotional regulation processes. These regions include the posterior cingulate cortex (PCC), which plays a critical role in mediating the default mode network (DMN) and driving self-reflective processing [15,16]; the OFC, which is involved in monitoring reward valuation and subsequent decision-making; and the hippocampus, which is primarily involved in episodic memory, and receiving and associating information [17]. Evaluating the rsFC of these brain regions may provide insight into how mental health and substance use are associated with an athlete’s ability to regulate emotion. The present study explored associations between substance use, mental health symptoms, and the rsFC of significant neural hubs involved in self-monitoring and emotional regulation in a sample of female varsity athletes. We hypothesized that substance use would correlate with increased symptoms of anxiety and depression in female athletes, and these behavioural differences would be reflected in underlying rsFC differences.

## Materials and methods

### Participants

Thirty-one female varsity athletes from York University, Toronto, between the ages of 18 and 25 years (*M* = 19 years 9 months, *SD* = 1 year 6 months) were recruited at preseason (i.e., prior to training camp) through the York University Sports Medicine Clinic and baseline data were collected. Participants were eligible if: i) they were over 18-years-old, ii) were rostered to play on a university team for the current season, and iii) met the requirements for safe MRI scanning. Three of the participants (9.6%) reported a history of prior mental health treatment and one indicated current treatment with anti-depressant medication (3.2%). Twenty-one of the participants (68%) reported current use of a hormonal contraceptive. Participants were predominately Caucasian (79%). A sample size of 31 was more than sufficient to detect an effect for planned comparisons, based on a calculation using 80% power, a medium effect size (0.5), and a p-value of 0.05 (N = 12) [18]. Participants were first active in sports by approximately six years of age (*M* = 6 years 2 months, *SD* = 2 years 11 months). At the time of the study, the athletes were engaged in one of the following sports: hockey (*n* = 13, 42%), volleyball (*n* = 6, 19%), soccer (*n* = 7, 23%), or basketball (*n* = 5, 16%). Ninety-four percent of participants (n = 29) were right-handed.

### Procedure

This study was approved by the Human Participants Review Sub-Committee of York University’s Ethics Review Board. Participants gave written informed consent to complete the imaging and behavioural components of the study, which were conducted on the same day. Participants were compensated $50 for their time.

### Measures

#### Patient Health Questionnaire-9 (PHQ-9)

The PHQ-9 [19] is a 9-item questionnaire of self-reported symptoms of depression. Participant total depression severity score is calculated by summing the items; total scores of 5, 10, 15, and 20 represent mild, moderate, moderately severe, and severe depression, respectively.

#### Generalized Anxiety Disorder-7 item (GAD-7)

The GAD-7 [20] is a well validated, 7-item questionnaire to assess self-reported anxiety. Total score cut-offs of 5, 10, and 15, represent mild, moderate, and severe anxiety, respectively.

#### Substance use

The Alcohol Use Disorder Identification Test-Clinical (AUDIT-C) screening index [21] was used to assess alcohol use. Total scores of 4 or more in men and 3 or more in women indicate hazardous or harmful drinking. Cannabis use was evaluated using a health questionnaire, which includes the question “how often do you smoke marijuana?” Possible responses to this question were never, monthly or less, 2–4 times a month, 2–3 times per week, 4+ times per week. Alcohol use was determined by a score of 3 or higher on the AUDIT-C and participants were considered cannabis users if they indicated any answer other than “never” to “how often do you smoke marijuana?” on the health questionnaire. Using these criteria, participants were divided into non-users and users. Users were further divided into alcohol users (alcohol but not cannabis use), and cannabis users (cannabis use regardless of alcohol use).

#### Sport Concussion Assessment Tool-5 (SCAT5)

The SCAT5 is a widely used standardized tool for assessing concussion which combines the Standardized Assessment of Concussion (SAC), the Post-Concussion Symptom Scale (PCSS), and on-field signs of concussion [22].

### MRI acquisition

Using a Siemens TIM Trio 3T MRI scanner (Siemens, Erlangen, Germany), T1-weighted, high-resolution anatomical scans were acquired using ascending multi-slice 3D Magnetization Prepared Rapid Acquisition Gradient Echo (MP-RAGE) (FOV = 256 mm, 1.0 x 1.0 x 1.0 mm voxels, TR = 2300, TE = 2.62 ms). A whole brain multi-echo echo-planar imaging (EPI) sequence, which is T2*-weighted, was used to acquire functional data in 240 volumes (43 slices, FOV = 216 mm, 64 x 64 matrix, 3.4 x 3.4 x 3.0 mm^3^ voxels, TR = 3000 ms, echo times [TEs] = 14 ms, 30 ms, 46 ms, flip angle = 83°). During fMRI scanning, participants lay still with their eyes closed.

### Functional imaging preprocessing

Functional imaging data (i.e., the second echo from the multi-echo scan) were preprocessed and analyzed using the CONN toolbox Version 18.b [23] using a standard preprocessing pipeline in which functional data were functionally realigned and unwarped by estimating and correcting the change in the distortion map with respect to the motion parameters, translated by centering to (0,0,0) coordinates, slice-time corrected, scrubbed with ART-based identification for outlier scans, segmented into grey matter (GM), white matter (WM), and cerebrospinal fluid (CSF), normalized to the Montreal Neurological Institute (MNI) template MNI152, and smoothed using an 8 mm Gaussian kernel, full width at half maximum [24]. The toolbox’s aCompCor protocol was used to reduce physiological and movement effects (i.e., signal from white matter and, cerebrospinal fluid, outliers detected by the ART toolbox, and realignment parameters from preprocessing were entered into the linear regression as confounding effects). A band-pass filter (0.008–0.09 Hz) was applied to the data.

### Statistical analysis

Variables were examined for normality and univariate outliers; there were no violations to assumptions of normality and therefore, parametric tests were used. T-tests and Hedge’s g (effect size) were conducted between users and non-users and alcohol and cannabis users to evaluate differences in age, age at first sport, concussion history, SCAT symptoms, SAC scores, depression and anxiety symptoms. Follow-up analyses were performed between non-users and alcohol users, and non-users and cannabis users.

### ROI-to-ROI analysis

CONN toolbox ROI-to-ROI analyses were used with weighted General Linear Model (GLM) correlation analysis settings (described in detail elsewhere) [23]. Using the default atlas in CONN toolbox, source seeds selected included the PCC (x = 1, y = -37, z = 30), the left and right hippocampus (Left, x = -25, y = -23, z = -14; Right, x = 26, y = -21, z = -14), and the left and right OFC (Left x = -30, y = 24, z = -17; Right x = 29, y = 23, z = -16). All atlas seeds (132) were selected as targets to the analysis. ROI-to-ROI matrices represent the level of functional connectivity between each pair of ROIs (i.e., all pairwise correlations of BOLD signal time-series between each seed and all targets). Each element is defined as the Fisher-transformed bivariate correlation coefficient between a pair of ROI BOLD time-series [25]. Between-subjects contrasts were run comparing [Users (1) non-Users (-1)] and [Cannabis Users (1) Alcohol Only Users (-1)]. A within-group follow-up analysis was run investigating the effect of depression in cannabis users using demeaned PHQ-9 scores. ROI-to-ROI rsFC was examined using intensity-based thresholding set to a False Discovery Rate (FDR) of p ≤ 0.05 seed-level correction, two-sided.

## Results

From the sample, 21 participants were classified as substance users and 10 were classified as non-users. Among the substance users, 13 used alcohol only, and 8 reported concurrent cannabis use. There were no significant differences between users and non-users in age, age of first sport, concussion history, SAC scores, or GAD-7 scores. However, users had significantly higher PHQ-9 scores [t(29) = -2.165, *p* = 0.039, Hedge’s g = 0.81; large effect] and higher SCAT symptom total scores [t(29) = -2.106, *p* = 0.04, Hedge’s g = 0.79; large effect] (See Table 1). PHQ-9 and SCAT symptom totals were significantly higher when comparing the non-users and cannabis users (p < .05) but not alcohol only users (see Table 1). There were no statistically significant differences in clinical characteristics between alcohol only users and cannabis users except in PHQ-9 scores where cannabis users had significantly higher scores [t(19) = 2.759, *p* = 0.012, Hedge’s g = 1.20; large effect].

**Table 1:** Participant characteristics.

	Non-Users (n = 10)	Users (n = 21)	p(g)	Alcohol (n = 13)	p(g)^a^	Cannabis (n = 8)	p(g)^b^
Age *M(SD)*	19.7 (1.42)	19.8 (1.54)	0.8 (0.07)	19.54 (0.97)	--	20.2 (2.19)	--
Age at First Sport *M(SD)*	5.6 (2.67)	6.38 (3.04)	0.49 (0.26)	7.00 (3.37)	--	5.38 (2.26)	--
Concussion History *Md(Range)*	1.0 (0–4.0)	1.0(0–6.0)	0.94 (0.03)^c^	0 (0–3.0)	--	1.5 (0–6)	--
SCAT Symptoms Total *M(SD)*	1.40 (2.84)	4.57 (4.32)	**0.04 (0.79)**	3.38 (3.20)	0.14 (0.63)	6.50 (5.37)	**0.004 (1.50)**
SAC Total Score *M(SD)*	26.6 (1.35)	27.24 (1.30)	0.22 (0.47)	26.92 (1.19)	--	27.75 (1.39)	--
PHQ-9 Total *M(SD)*	1.60 (2.8)	4.14 (3.17)	**0.04 (0.81**)	2.85 (2.48)	0.27 (0.46)	6.25 (3.15)	**0.019 (1.17)**
GAD-7 Total *M(SD)*	1.70 (3.02)	3.29 (2.85)	0.17 (0.53)	2.77 (3.22)	--	4.13 (2.03)	--

Note: g = Hedge’s g;

^a^Comparison between non-users and alcohol users;

^b^Comparison between non-users and cannabis users;

^c^Mann-Whitney U Test to examine differences between distributions.

### ROI-to-ROI results

**Fig 1:**
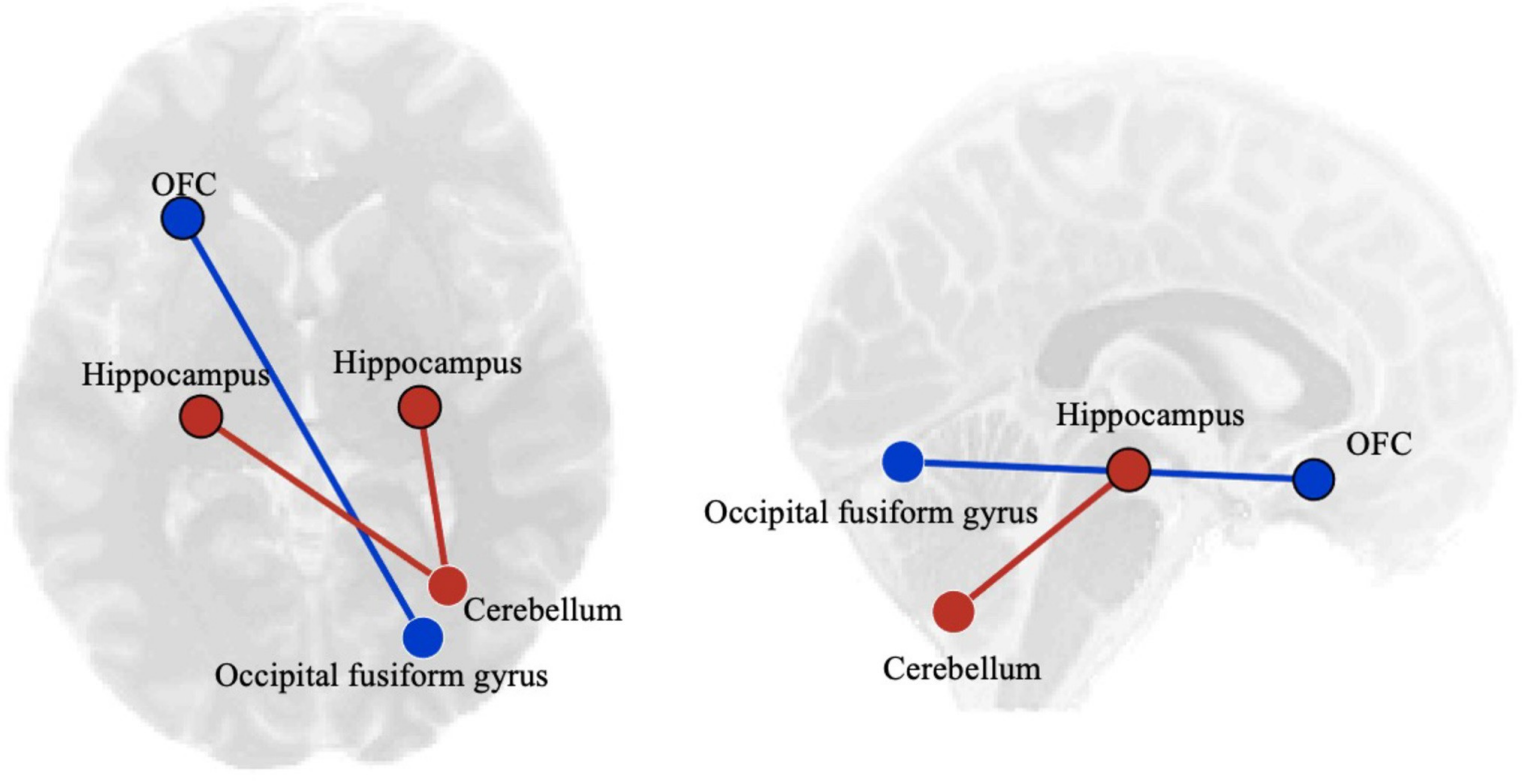
Differences between substance users and non-users showing increases (red) and decreases (blue) in resting state functional connectivity in substance users displayed from an axial view on the left and a sagittal view on the right. See Table 2 for ROI results. L, left; R, right.

**Fig 2:**
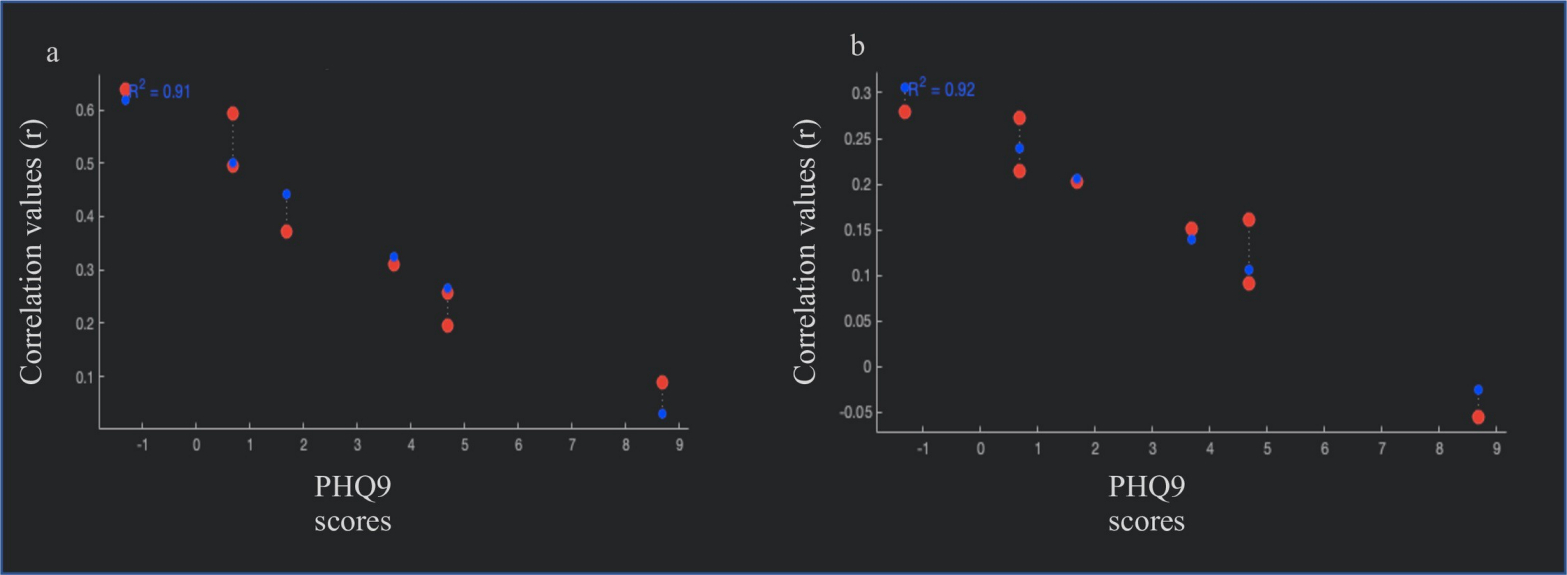
Correlation between demeaned PHQ9 scores and functional connectivity between (a) the fusiform cortex and hippocampus and, (b) the cerebellum and the posterior cingulate. Note: red dots = observed values; blue dots = fitted values.

Results from the ROI-to-ROI analyses are summarized in Table 2 and shown in Fig 1. Users showed decreased rsFC between the left OFC and the occipital fusiform gyrus (p = 0.032) and increased rsFC between both hippocampal seeds and the right cerebellum (ps ≤ 0.05) compared to non-substance users. When breaking down the user group into alcohol and cannabis users, no significant differences in rsFC were found between cannabis use and alcohol use only participants (ps > 0.05). To further examine potential associations between rsFC and PHQ-9 scores in cannabis users, an exploratory within-group correlation was conducted. PHQ-9 scores were negatively correlated with rsFC between the left hippocampus and the right posterior temporal fusiform cortex (p = 0.020), and between the PCC and the cerebellum (p = 0.027; See Fig 2).

**Table 2:** ROI results.

Contrast	Seed	Target	Target MNI coordinates			T	pFDR	Connectivity r-values	
			x	y	z			Users *Mean(SD)*	Non-Users *Mean(SD)*
Users > Non-Users									
	OFC left	Occipital fusiform gyrus right	-27	-77	-14	-4.18	0.032	-0.04(0.12)	0.14(0.09)
	Hippocampus right	Cerebellum right	33	-63	-48	4.44	0.016	-0.03(0.10)	-0.21 (0.12)
	Hippocampus left	Cerebellum right	33	-63	-48	4.11	0.038	0.01(0.12)	-0.18(0.13)
Effect of PHQ-9 for Cannabis Users									
	Hippocampus left	Posterior temporal fusiform cortex right	36	-24	-28	-8.42	0.020	-0.96^a^	--
	PCC	Vermis45	1	-52	-7	-7.97	0.027	-0.96^a^	--

Note: OFC = orbitofrontal cortex; PCC = posterior cingulate cortex; PHQ-9 = Patient Health Questionnaire-9.

^a^This reflects the r value of the regression.

## Discussion

The present study showed that within a relatively small female university athlete sample, approximately two thirds (21/31; 67.7%) endorsed moderate alcohol use and/or combined alcohol and cannabis use. Combined substance users also endorsed significantly greater symptoms of depression and total symptoms on the SCAT5. Prior systematic reviews have reported that cannabis use, and heavy cannabis use, in particular, is associated with elevated risk of depression [26] and negative affective outcomes (e.g., depression, suicidal thoughts, and anxiety) [27]. Furthermore, co-use of alcohol and cannabis has been associated with greater risk of comorbid mental health disorders [28]. Our findings demonstrate increased symptom endorsement (i.e., of depressive and other psychological and somatic symptoms) in female athletes with self-reported cannabis use compared to alcohol only users and non-users. Concussion history has also been associated with higher alcohol and cannabis use in college students [29]. Athletes are at greater risk for concussion [30], and prior research has suggested that higher exposure to repetitive head injuries is associated with increased substance use [31] and symptoms of depression [32,33]. Higher exposure to repetitive head impacts has been suggested to affect vulnerable brain areas involved in impulsive and risk-taking behaviours, which may account for increased substance use [31]. Within our limited sample size, we did not observe differences in concussion history between substance users and non-user; however, the relationship between cannabis use and symptom endorsement in athletes requires further investigation to explore whether associations between substance use and mental health status are mediated by concussion history.

This study also examined associations between substance use and rsFC in regions of the brain involved in emotion processing and regulation. Relative to non-users, athletes who used alcohol or cannabis exhibited hypoconnectivity between the left OFC and the occipital fusiform gyrus while showing hyperconnectivity between the bilateral hippocampus and the right cerebellum. Within the cannabis users, depression symptoms correlated negatively with connectivity of the left hippocampus and the PCC. These associations suggest that alterations in rsFC exist in substance-using athletes relative to their non-using athlete peers. Emotion and hippocampal networks of the limbic system are involved in reward-related decision making [17] and altered connectivity patterns within these networks may dysregulate decision-making abilities with implications for risky substance use behaviour.

To our knowledge, this is one of the first studies examining substance use (in particular cannabis use) and its assocations with mental health and neural functioning in athletes at baseline. Despite the limited sample size, this study found associations between substance use, mental health, and underlying brain functional connectivity in self-monitoring and emotional regulation regions in female varsity athletes (with moderate to large effect sizes). These preliminary findings highlight the need to better understand relationships between substance use and mental health in athletes. However, the measurement of substance use was limited in this study and future research should further examine associations between cannabis use, affective disorders, and concussion history in athlete populations using more comprehensive measures of substance use. In addition, further research is necessary to examine whether differences in rsFC connectivity within default mode and limbic networks affects self-monitoring and emotional regulation that may, in turn, influence substance use behaviour. This work suggests a need to consider factors such as mental health and stubstance use when examining neural functioning of athletes at baseline and, potentially, post-intjury. It also highlights the importance of assessing substance use and monitoring of mental health in athletes. Further research in the area may lead to the development targeted interventions to improve outcomes for athletes.
